# Supplementary figures and images for: The relationship between PLOD1 expression level and glioma prognosis investigated using public databases
Source: PeerJ. 2021 May 14;9:e11422. doi: 10.7717/peerj.11422 (PMC8127981; doi:10.7717/peerj.11422)

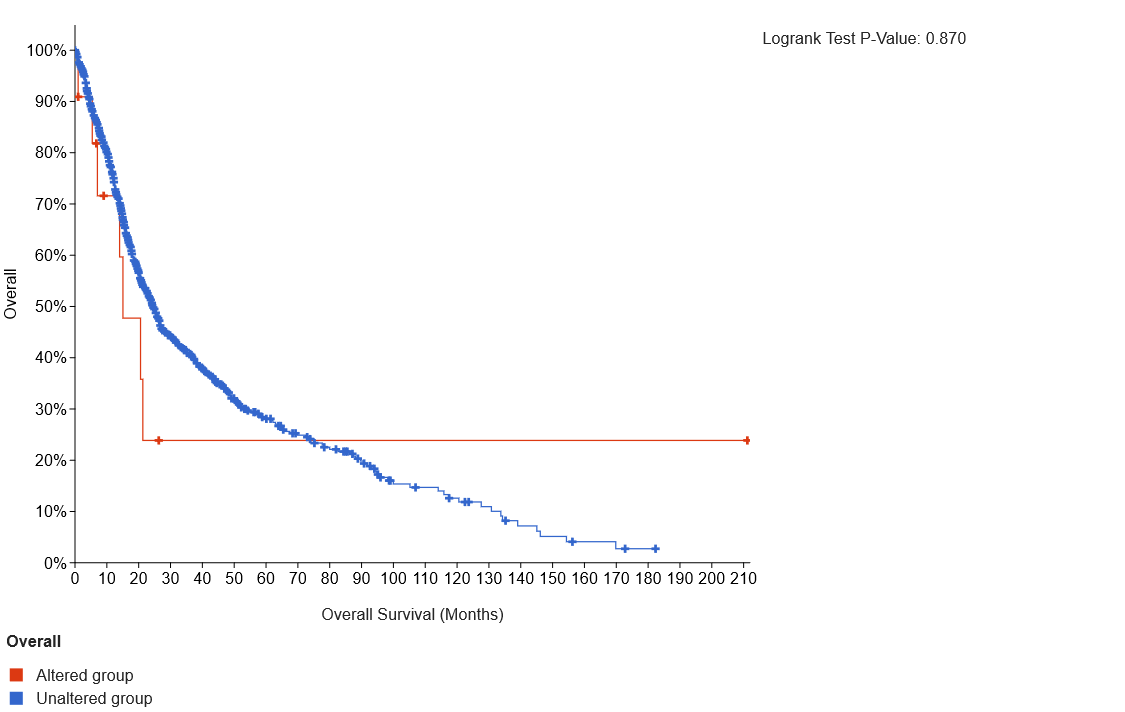

Supplement: Supplemental Information 12 [file peerj-09-11422-s012.png]
